# Engaging With a Web-Based Psychosocial Intervention for Psychosis: Qualitative Study of User Experiences

**DOI:** 10.2196/16730

**Published:** 2020-06-19

**Authors:** Chelsea Arnold, Anne Williams, Neil Thomas

**Affiliations:** 1 Centre for Mental Health Swinburne University of Technology Hawthorn Australia; 2 School of Health Sciences Swinburne University of Technology Melbourne Australia; 3 Department of Psychology and Couselling La Trobe University Melbourne Australia; 4 The Alfred Hospital Melbourne Australia

**Keywords:** psychosis, engagement, qualitative research, eHealth, internet intervention, mobile phone

## Abstract

**Background:**

Web-based interventions are increasingly being used for individuals with serious mental illness, including psychosis, and preliminary evidence suggests clinical benefits. To achieve such benefits, individuals must have some level of engagement with the intervention. Currently, little is known about what influences engagement with web-based interventions for individuals with psychotic disorders.

**Objective:**

This study aimed to explore users' perspectives on what influenced engagement with a web-based intervention for psychosis.

**Methods:**

A qualitative design was employed using semistructured telephone interviews. Participants were 17 adults with psychosis who had participated in a trial examining engagement with a self-guided, web-based intervention promoting personal recovery and self-management of mental health.

**Results:**

We identified 2 overarching themes: *challenges to using the website* and *factors supporting persistence*. Both of the main themes included several subthemes related to both user-related factors (eg, mental health, personal circumstances, approach to using the website) and users’ experience of the intervention (eg, having experienced similar content previously or finding the material confronting).

**Conclusions:**

Individuals with psychosis experienced several challenges to ongoing engagement with a web-based intervention. Adjunctive emails present an important design feature to maintain interest and motivation to engage with the intervention. However, fluctuations in mental health and psychosocial difficulties are a significant challenge. Design and implementation considerations include flexible interventions with tailoring opportunities to accommodate changeable circumstances and individual preferences.

## Introduction

### Background

Access to the internet and digital technologies is increasing among individuals with psychosis [1,2]. Many individuals with psychosis are active and proficient users of technology and the internet [3-5] and demonstrate positive attitudes toward using digital technologies to support mental health and self-management [6,7]. This increased access and interest provide a unique opportunity to promote self-management of mental health via internet- and mobile-based interventions. To meet this opportunity, several mobile apps and internet-based interventions have been developed to promote self-management of mental health in psychosis [8-11]. Both internet- and mobile-based interventions (hereafter named *web-based interventions*) appear feasible, acceptable, and potentially effective in improving clinical outcomes for individuals with psychotic disorders [12-15].

Despite potential benefits, engagement with web-based interventions for psychosis may be variable [16]. Some individuals may be very engaged and high users of an intervention. However, a large proportion will have only initial or infrequent use of an intervention, thus limiting potential associated benefits [17]. Individuals with psychotic disorders may have specific difficulties related to engaging with web-based interventions. For example, cognitive deficits, reduced awareness of illness, and positive and negative symptoms may present as challenges to using the internet and web-based interventions [4,18]. Additionally, some individuals with psychosis may have limited computer literacy and additional barriers to internet use and access. These barriers are potentially associated with less educational and vocational attainment or the impact of symptoms [2,4,19].

Qualitative methods can enable the exploration of user experiences and reveal factors that support or obstruct engagement with web-based interventions [20]. The investigation into web-based interventions for broader mental health difficulties has highlighted various factors that impede or support engagement with web-based interventions. Previously identified challenges to engagement include mental health symptoms (such as low mood, anxiety, and mania), current life situation (such as competing demands), and negative perceptions or experiences of the intervention (eg, perceived poor fit with the user) [21-25]. Conversely, positive intervention experiences (such as a sense of autonomy, competence, or connection) and finding an intervention beneficial have previously been reported as facilitators of engagement with web-based interventions [21,26,27].

Previous research has qualitatively investigated users’ experiences of web-based interventions for psychosis [28-30]. However, these studies have largely focused on broader perceptions and evaluations of relevant interventions, such as acceptability and associated impacts. To date, limited research has specifically examined how people with psychosis engage with web-based interventions or factors that may influence engagement. Eisner and colleagues [10] recently investigated barriers to engagement with a mobile app for symptom monitoring in schizophrenia (the Experiences of Psychosis Relapse: Early Subjective Signs app). Participants who used the app over 6 months identified both mental health and phone-related barriers to engagement with the app. Some participants reported positive symptoms impacted concentration, which made answering questions on the app more strenuous. Others reported difficulties using smartphones because of a lack of experience.

### Objectives

Web-based–self-management programs are another type of digital intervention for which there is a lack of research examining user experiences of engagement. This study aimed to explore user experiences of engaging with a web-based program for psychosis and identify factors associated with engagement and disengagement.

## Methods

### Research Context

This qualitative study was embedded in a broader research project that examined predictors of engagement with a web-based intervention for individuals with psychosis—the Self-Management and Recovery Technology (SMART) website [16]. The broader research program involved a trial on the use of the website on a tablet computer in face-to-face sessions to examine efficacy [31,32] and examining integration into routine mental health services. This study formed part of an investigation into the potential to use the SMART website as a predominantly self-guided, low-intensity, web-based intervention [16].

**Figure 1:**
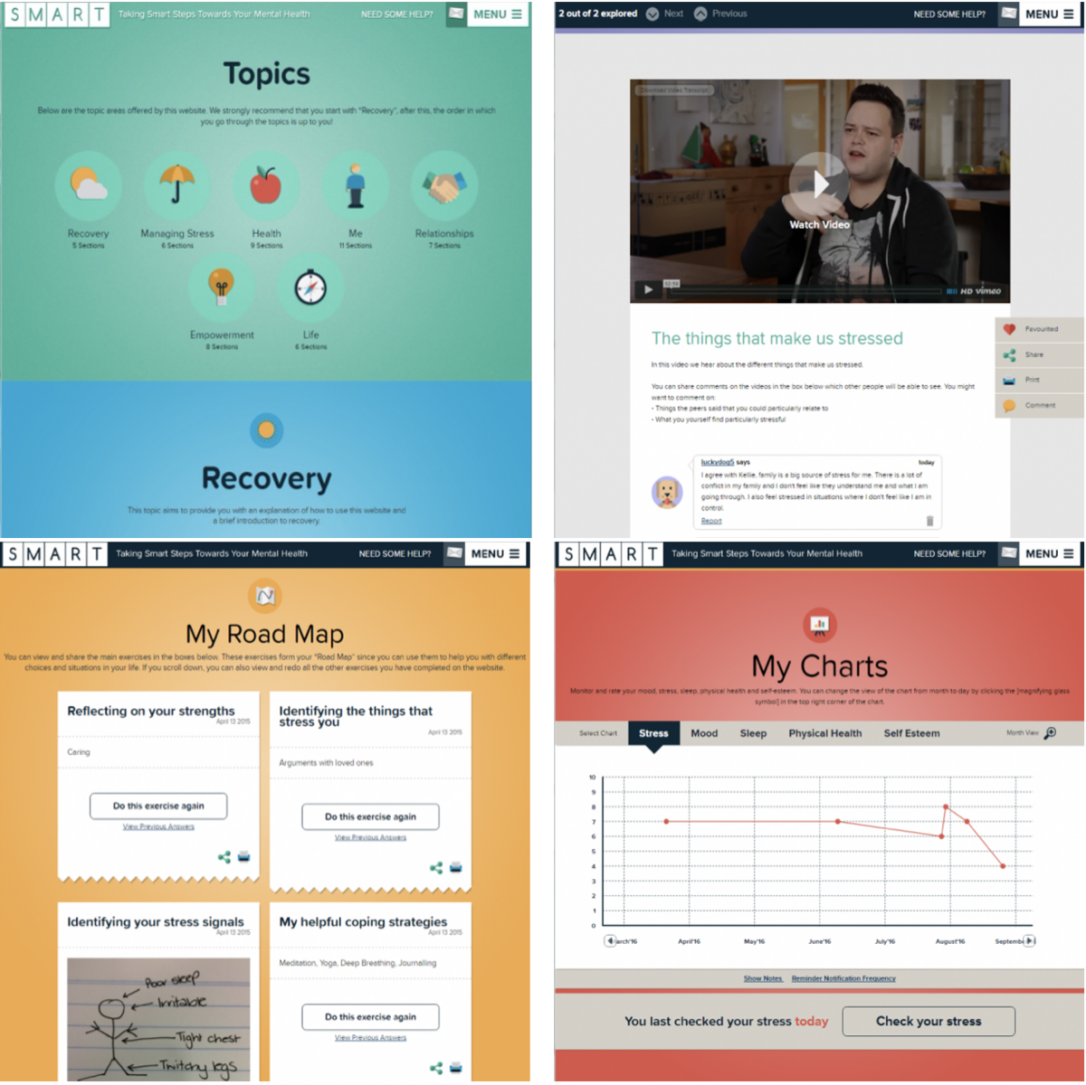
Example screenshots from the Self-Management and Recovery Technology website.

The website aimed to promote self-management of mental health and personal recovery (ie, living a meaningful and satisfying life despite potential difficulties associated with experiences of mental illness) in people with psychotic disorders [31,33]. Website content was organized into 7 modules on recovery, stress management, relationships, health, values, empowerment, and stigma and identity. Each module contained video-based content of peers with lived experience of psychosis, discussing their personal experiences and opinions of the particular topic and associated activities. Additional website features included charting tools (eg, to track stress, sleep, etc) and social networking features, including a peer-moderated forum (Figure 1 shows example screenshots).

The website was designed in accordance with guidelines for individuals with serious mental illness and cognitive deficits [34]. This included simplification of navigation and content and minimization of text where possible [31]. Website development involved a co-design process with consumers and mental health workers who indicated a preference for an intervention that could be used flexibly rather than sequentially. Additionally, as this resource focused on personal recovery, a highly individualized process [35], the website was designed as a resource of web-based materials that could be used flexibly. The *Recovery* module included information on how to use the website and was the recommended but optional starting point. Otherwise, the website did not have a specified order or end point and could be used in accordance with users’ personal preferences. A full description of the SMART website is detailed elsewhere [31].

A total of 98 participants were recruited from across Australia and given access to the website for ≥12 weeks. The website automatically randomized participants on a 1:1 basis to receive either (1) access to the website only or (2) access to the website in conjunction with weekly, asynchronous, support emails from a mental health worker at a project partner for 12 weeks. Support emails did not aim to be therapeutic but encouraged users to work through website material [16].

All participants completed research-related questionnaires before signing up to the website and received email reminders to complete follow-up questionnaires at 4 and 12 weeks postregistration. All participants could also select to receive additional system-automated reminder emails to complete activities on the website (eg, mood tracking). Participants used the website on their personal or otherwise internet-accessible devices. Users were advised to use the website as they chose in accordance with personal interests and preferences.

### Research Design

We employed an exploratory qualitative study using an inductive approach [36]. Semistructured interviews were used to enable a more in-depth discussion of experiences and provide opportunities to explore and clarify participant responses [37,38]. We used a semantic approach to theme development within an essentialist/realist paradigm aimed at objectively capturing the lived experience of participants [36,39].

### Participant Recruitment

Participants were drawn from the primary study [16]. The original inclusion criteria were the following: (1) aged between 18 and 65 years inclusive, (2) currently residing in Australia, (3) diagnosis of a nonorganic psychotic disorder (schizophrenia-related disorder, bipolar disorder, or major depressive disorder with psychotic features present within the past 2 years) confirmed using the Structured Clinical Interview for Diagnostic and Statistical Manual of Mental Disorders, fourth edition,-text revision Axis I Disorders [40], (5) proficiency in the English language, and (6) willingness to access and use the internet. Exclusion criteria included experiences of psychosis only during substance intoxication and no internet access.

Following the 12-week intervention period, participants were contacted via email by the first author (CA) and invited to participate in the interview. To minimize recall issues without impacting participants’ use of the website for the primary study, we aimed to conduct interviews as close as possible to the intervention end date. Initially, a pool of consecutive participants was invited to participate. After initial interviews, we purposefully selected participants with the goal of ensuring diversity of age, gender, condition (with and without adjunct email support), and use of the website (from no use to long-term, high use). We recruited participants until saturation occurred, and no new information arose from interviews. Participants were reimbursed with an AUD $30 (US $19.3) gift card for completing the interview. Some participants were previously assessed for eligibility for the main study by the interviewer (CA). Otherwise, there was no existing relationship between the interviewer and participants.

### Data Collection

Data were collected via semistructured telephone interviews with 17 participants. The interviews followed a semistructured interview guide with open-ended questions about potential factors that may have influenced engagement with the website (Multimedia Appendix 1). A panel of individuals with lived experiences of psychosis provided feedback on the interview guide before data collection began. During data collection, additional questions were formulated and added to the interview schedule in an iterative process, depending on participant responses. The interview schedule thus evolved over the course of the study. Field notes were made following each interview, and interviews were transcribed verbatim from audio recordings. Transcription of one interview was not possible because of a recording error. On this occasion, attempts were made to outline the interview content as closely as possible from memory.

### Data Analysis

Data were analyzed using a thematic analysis approach [36]. Data collection and analyses occurred concurrently. The first author familiarized herself with the data by listening to and reading each interview multiple times with reference to field notes. The first author initially coded all interviews using an inductive approach. The second author (AW) also coded early interviews and subsequently discussed the first author’s coding in the process of peer review. Following the completion of data collection and preliminary coding of all interviews, the first author recoded all data with new codes using NVivo 12 (QSR International) and then developed a coding framework. The coding framework was used to inform the development of categories and subsequent themes based on patterns identified in the codes. Developing categories and themes were discussed between the authors and refined in an iterative manner.

### Methodological Integrity

The authors were from clinical psychology (CA and NT) and occupational therapy (AW) professional backgrounds. The first author was familiar with the literature on engagement with web-based interventions among individuals with serious mental illness. Despite aiming to follow a data-driven approach, this knowledge may have influenced the interpretation of data. The first author’s perspectives were managed by reflective note taking and discussion with other authors to ensure that the interpretations were aligned with the collected data and not based on pre-existing assumptions. The first author received training in qualitative research methods. The second and third authors additionally had experience using qualitative research methods with individuals with psychosis. A panel of individuals with lived experiences of psychosis provided feedback on the authors’ understanding of data during thematic analysis and supported the interpretation. We followed the American Psychological Association reporting standards for qualitative research throughout this manuscript [41].

## Results

### Participant Details

A total of 17 participants were interviewed via telephone, with interviews lasting between 17 and 58 min (average time 30 min). There was a near equal distribution of participants who received access to the website only (n=8) and those who additionally received the support emails (n=9). Participants reported using the website on their smartphones, computers (desktops and laptops), tablet computers, and a combination of devices. Participants logged on to the website an average of 15 times (range 3-40) and completed an average of 62 activities on the website (range 6-316). Participant characteristics are outlined in Table 1.

**Table 1:** Participant characteristics (N=17).

Variable		Participants, n (%)	
**Age (years)**			
	18-34		7 (41)
	35-50		6 (35)
	51-65		4 (24)
**Gender**			
	Female		11 (65)
	Male		6 (35)
**Level of education**			
	Secondary school		5 (29)
	Apprenticeship or diploma or certificate		4 (24)
	Bachelor’s degree		5 (29)
	Postgraduate degree		3 (18)
**Recent employment status**			
	Paid or self-employment		4 (24)
	Unemployed		13 (76)
**SCID^a^** **diagnosis**			
	Schizoaffective disorder		5 (29)
	Schizophrenia		5 (29)
	Bipolar disorder with psychotic features		4 (24)
	Major depression with psychotic features		3 (18)
**Confidence using the internet**			
	Confident without assistance		15 (88)
	Occasionally need assistance		2 (12)
**Frequency of internet use**			
	More than once a day		16 (94)
	A few times a week		1 (6)

^a^SCID: Structured Clinical Interview for Diagnostic and Statistical Manual of Mental Disorders, fourth edition, text revision Axis I Disorders.

### Salient Themes

**Figure 2:**
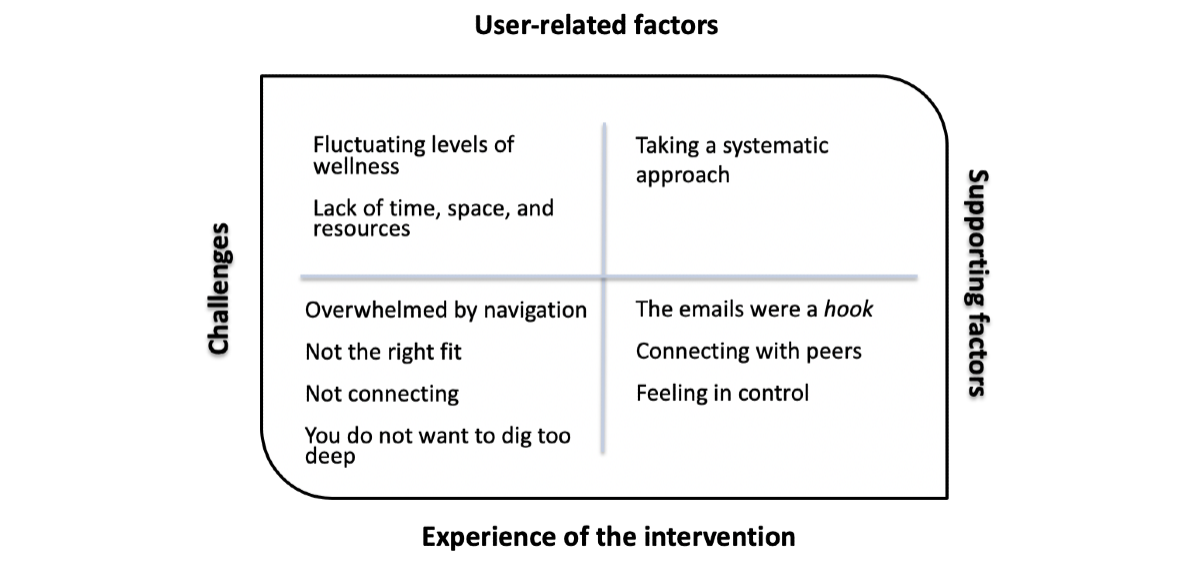
Challenges to engagement and factors supporting persistence with the website.

We identified 2 central themes related to participants’ engagement with the website: *challenges to using the website* and *factors supporting persistence*. Many participants spoke about initial interest and motivation to use the website and expected to use the resource more than they did. These participants identified significant challenges in engaging with the website. A smaller number of participants persisted with the intervention and reported a positive experience. They identified factors that supported them to sustain their engagement with the website. Each theme had subthemes relating to both user-related factors and users’ experiences of the intervention. The main themes and subthemes are outlined in Figure 2 and further discussed in the following sections. Quotes from participants have been included to illustrate each finding. Participants’ names have been replaced with a unique identifier to maintain participant anonymity.

### Challenges in Using the Website

#### User-Related Factors

##### Fluctuating Levels of Wellness

Fluctuations in participants’ mental health played an important role in their engagement with the website. A total of 9 participants reported mental health–related challenges to engagement. Participants referred to fluctuating levels of wellness that influenced their interest toward and the potential to engage with and gain meaning from using the website:

Because it's such a tidal wave of different mental states and sometimes normality, it really depends on the day… how you're feeling and how you can cope.

P17, website only

Participants spoke about motivation difficulties associated with psychosis and mood difficulties. During periods of low energy and motivation, the efforts of participants were focused on tasks of daily living. As such, anything *extra*, such as the website, was not a priority or deemed too burdensome to consider:

And it's really hard to get motivated to do anything. And that was sort of like the last thing, you know? Like getting up is hard enough. Let alone trying to [use the website].

P14, email support

Paranoid thoughts also impacted participants’ willingness to engage with the website. A total of 3 participants reported fluctuating levels of suspiciousness toward technology and concerns about the privacy of the information on the web:

I personally don't feel comfortable typing any of the answers to any of these things into a website. Because, part of my paranoid thoughts is this feeling like, issues about privacy and technology. And it’s just all too much for me to type.

P10, website only

A participant mentioned not using the website during a psychotic episode that she experienced when participating in the study. The participant reported that during periods of psychotic *relapse*, she did not use the intervention as she would have had difficulty processing the material on the website:

I guess I just can't really stand the screen and words get jumbled up and things like that… I wouldn't have been able to make sense of the topics…

P7, website only

This was seconded by another participant who highlighted the limited benefit of resources such as the website during a psychotic episode:

I know that this SMART online wouldn't have helped me then…. It's like you won't even come to the website. And, even if someone puts you in front of the website, it just would not mean anything.

P11, website only

Importantly, when the impact of symptoms decreased, some participants were able to re-engage with the website.

##### Lack of Time, Space, and Resources

Engagement with the SMART website was influenced by the personal circumstances of participants. A total of 7 participants reported struggling to find time to spend on the website or simply forgot:

Time was an important factor. I just didn't have the time that I'd like to spend on it. And, I couldn't get into it as thoroughly as I would have liked.

P5, email support

Overall, 5 participants experienced significant psychosocial stressors during their participation period, including periods of homelessness, physical health difficulties, and accidents. As highlighted by a participant, a period of homelessness meant that the website was not a priority for him:

… around late February my living situation changed drastically. And so, yeah, I no longer have the time or space to do it.

P13, email support

For 4 participants, gaining access to the website was also a practical barrier to engagement. Some participants did not have ready access to the internet because of financial limitations or homelessness, and others had limited data on their mobile phones. As the majority of participants had only 1 device available to them, this meant that participants did not have the means to access the website.

#### Experience of the Website

Once a participant had accessed the website, their early experience of the website contributed to their decision making about continued engagement with the website or disengagement. In particular, whether or not participants persisted with using the website was influenced by experiencing difficulties navigating the site, a lack of connection with the content or users, and negative emotional experiences associated with use.

##### Overwhelmed by Navigation

Overall, 5 participants found it difficult to navigate the website. When experiencing this difficulty, some participants felt that using the website required too much effort or was overwhelming:

I saw that there was forums and stuff. And I wanted to go and do it, but then I just couldn't navigate it. And I was just like, “Oh, I can't be bothered.”

P14, email support

For others, the lack of direction in how to use the website, in combination with the amount of content, was daunting and overwhelming:

It felt like because it's all listed there in sections and there's so many there that it felt like you try and start reading them you feel like you're having a bit of trouble getting through it. And then you think, “Oh well, I might as well just not bother trying to read all these, or watch all these, because it's all a bit much.”

P10, website only

##### Not the Right Fit

Early experiences of the intervention influenced the interest participants had in persisting with the intervention. When participants had previously encountered similar material, or they deemed the website irrelevant to their immediate needs, their interest in continuing waned:

Either it wasn't relevant or it was stuff I'd sort of thought about previously already. So I didn't feel I needed to go over it again.

P8, email support

##### Not Connecting

A total of 8 participants reported signing up to the SMART website because they wanted to hear from, learn about, and share with individuals with similar experiences:

I was interested in the forums and to see what other people were saying about their experiences with mental health and hearing voices. And to see if there was any similarities with my experience and other people, and to kind of connect.

P2, website only

When participants sought connections with others but did not have this experience as part of using the website, their motivation to continue using aspects of the website, such as the forum or the videos, decreased:

To be honest, I haven't connected with anyone on the SMART website… Nothing has jumped out at me, and I haven’t really gone in there and attempted to make contact… If I had to point to a negative, it would be the fact that it’s not social, it’s not socially enabled.

P11, website only

##### You Do Not Want to Dig Too Deep

Overall, 3 participants found some of the content of the website to be deflating, confronting, or triggering:

Depending on how you’re feeling, it can be quite triggering… and it kind of makes you fear the reality of this is really how you're feeling. Yeah, it kind of puts it into reality what's there.

P14, email support

For these participants, this was an unpleasant reminder about their situation. They preferred not to think about their mental illness, as it was associated with difficult emotions, as highlighted by 2 participants as follows:

Well, it's interesting. When you have a mental illness you don't like to dig too deep too often because it's quite depressing…I try to keep my mental illness in my pocket, if you like.

P17, website only

My best, my best, days are when I typically forget, um, that I have any kind of illness. And I get to live life for a little while without remembering that it's an issue that I actually face. And I get caught up in just everyday things that everybody else gets caught up in. And things seem normal like they were before I was sick. So to be reminded, or to be very heavily reminded about the gravity of it all can be a bit, unpleasant.

P10, website only

These unpleasant emotions resulted in an urge to withdraw from the website:

But I feel like it's the kind of content that for some reason that feeling of stigma just kicks in you know every couple of minutes and you start to feel like, “Well maybe, well maybe I shouldn't do this.”

P10, website only

### Factors Supporting Persistence

#### User-Related Factors

##### Taking a Systematic Approach

The few participants who persisted with the intervention over the 12-week period came up with strategies that supported their continued use of the website. For example, a participant spoke about incorporating the SMART website into her weekly routine and a methodical approach to using the website:

I try to get on there on Saturdays… I basically go to a topic that I haven’t completed and I try to complete the topic or I go to a new topic and I start with the videos. And then I go through the activities and fill in the activities.

P7, website only

Similarly, another participant who persisted with the website spoke about making her way through the website systematically:

I tried to do it systematically… I went through each section and first I did the main things not the extras. And then over time I did mark the extras and I got through most of the content.

P4, email support

#### Experience of the Website

##### The Emails Were a Hook

The majority of participants (n=15) reported finding the system-automated or support emails particularly helpful for the ongoing use of the website. As highlighted by the following participant, receiving the emails reminded participants about the website and prompted them to re-engage with the resource:

Having the prompts all the time like the emails was really good, because it sort of made me think, “Oh, you know, I should jump on and have a look and see if there's anything that I can find that might help me stay positive and keep my mental health going well while I'm sick.”

P16, email support

Additionally, the support emails from the web-based therapist served as an incentive for some participants to persist with the website. They not only reminded participants about the website but also made individuals interested in returning to the website and more invested in using the resource:

… I knew that [the web-based therapist] would be responsive no matter what I said. So, it kind of made me look forward to those email and sign back on to the website. It was like a hook.

P4, email support

Some participants felt a sense of connection with their web-based therapist. When individuals felt supported by their therapist, it facilitated connection and made participants more open to using the website:

It sort of made it feel a little more personalised, made me more open to accessing the service. Knowing that there was a real person… on the other end made a fair difference.

P13, email support

##### Connecting With Peers

Overall, 5 participants resonated with the experiences of their peers in the videos, or with other users on the forum. For those who did connect with the experiences of others, this shaped their experience of the website into a positive one. For some users, this connection facilitated the ongoing use of the website:

*It was really interesting to me. I really liked getting to know the different interviewees and following them through the different topics…* *I just was so motivated to see the videos. It's like meeting new people in the videos.*

P7, website only

##### Feeling in Control

A few participants (n=4) reported a strong appreciation for the flexibility of the intervention. They felt in control of their use of the resource. This was a change from previous experiences and resulted in a positive view of the resource. They reported appreciating this flexibility and even engaging more broadly as a result:

It was a lot more flexible than things I've dealt with in the past. I think I just looked into more things because they were readily available…. Yeah, it was never a case of it's either this way or the highway, it was a case of well, if this isn't great for you then try something else.

P3, email support

## Discussion

### Principal Findings

This study explored the experience of individuals with psychosis engaging with a web-based psychosocial intervention with and without adjunct email support. Participants who initially evaluated the website as novel and/or relevant to them, intended to continue using the website. Those who persisted with the website used strategies to support use and worked through website content systematically. Both the reminder and support emails were helpful factors in persisting or re-engaging with the resource for many participants. Other participants intended to persist with the intervention, but mental health difficulties, psychosocial stressors, and limited access served as barriers to engagement. Feeling overwhelmed when navigating the website or feeling dejected by the content deterred participants from engaging with the website. When participants initially evaluated the resource as being irrelevant or not useful, there was also limited interest in persisting with the resource.

### Leveraging Supportive Factors

In this study, receiving email support was a key facilitator of engagement. This is consistent with findings from the primary study, where individuals receiving adjunct email support were significantly more engaged than participants accessing the website only [16]. The qualitative findings assist in explaining this difference. As with previous research, participants who did not receive email support found that their motivation to persist with the intervention waned quickly [23,42]. In contrast, receiving support emails made participants more open to using the website and maintained interest and persistence over time. Some participants felt accountable to their email support person, suggesting that emails served as an effective external motivating factor to engage [43].

In addition to the support emails sent by the mental health worker, automated emails were also perceived as helpful. The automated emails reminded participants about the website and enabled re-engagement with the resource following periods of nonuse. This is consistent with the previous investigation, where automated emails increased adherence with self-guided interventions, and reminders have been regarded as especially helpful [44,45]. Personalized support emails appear optimal as they can promote motivation to engage and explore resources. However, periodic automated reminders may be a cost-effective approach to remind users about the intervention and promote engagement. 

Users who persisted with the website resonated with the content and connected with their peers in the videos or other website users. In contrast, participants had limited motivation to persist with the resource when it was perceived as irrelevant or nothing new. Relatedness (ie, recognition and connection) is considered an important factor in promoting intrinsic motivation to engage with interventions [46]. Community, connection, and support opportunities have largely been experienced as positive components of web-based interventions [44,47]. Our findings support the importance of relatedness and the inclusion of social opportunities in intervention design. Additionally, tailoring interventions to accommodate varying interests may be helpful to ensure that interventions are relevant and accordingly promote motivation and engagement [48,49].

The results of this study and previous research suggest that a sense of control is likely to promote engagement with web-based interventions [21,27]. Interventions may therefore seek to incorporate ways to promote autonomy throughout [27]. This could include, for example, options for self-tailoring and options for choice [50]. This may be especially important for individuals with psychosis who may have had negative and disempowering mental health–related experiences, such as involuntary treatment [51].

Finally, individuals who persisted with the intervention used various strategies to support their use of the intervention. Incorporating goal setting to support the implementation of the website into the lives of users may therefore be a helpful component to promote engagement [52].

### Overcoming Challenges

Consistent with reports from other diagnostic groups, mental health appears to be an important factor in engagement with web-based interventions for psychosis [24,25]. Participants with and without adjunct email support reported that fluctuations in mental health impacted engagement. Individuals with psychosis have previously reported reduced internet use when unwell because of motivational and comprehension difficulties [53]. Concurrently, some participants in this study reported low mood and energy decreased motivation to engage with the website. Other participants reported suspiciousness toward the intervention, which resulted in nonuse. Additionally, participants regarded web-based interventions as having limited potential utility during episodes of psychosis. These results support a recent finding that mental health symptoms interfere with user engagement with a mobile monitoring app for psychosis [10]. Importantly, when the impact of symptoms decreased, some participants re-engaged with the website following receipt of reminder emails.

Participants reported difficulties in finding time to use the website. This has been identified as a challenge in previous studies of web-based interventions for other mental health difficulties such as depression, anxiety, and bipolar disorder [21,42,54]. Several participants in this study also faced significant psychosocial stressors such as homelessness during the period of participation. This has not commonly been reported among other diagnostic groups and suggests that this may be a unique challenge for some individuals with psychosis. During periods of acute psychosis or crisis, needs and priorities may shift from long-term recovery goals to one’s immediate situation and safety [55]. Although email support generally promoted engagement in this study, it did not appear to overcome serious mental health or psychosocial difficulties. Rather than advocating for continued engagement, web-based interventions may need to enable flexible use over time, depending on current needs and circumstances. A flexible resource that can accommodate periods of nonuse and allow for re-engagement when circumstances and mental health permits may, therefore, be optimal.

Individuals with psychosis and broader mental health difficulties have reported challenges in using the internet and web-based interventions because of low levels of computer skills and confidence [10,26,53]. Despite attempts to simplify website design and content, some participants in this study experienced difficulty navigating the website and found this process overwhelming. This finding supports the design of interventions that are simple, intuitive, and easy to use to accommodate potential lower levels of computer literacy [56]. A co-design process with both consumers and human-computer experts may serve to improve the design and usability of web-based interventions [50,57]. More on-demand technical support or initial training in how to use an intervention may be helpful to overcome this difficulty [18,58].

Some participants found website content confronting and upsetting, which discouraged them from persisting. This mirrors reports from individuals with bipolar disorder who disengaged from a previous web-based psychoeducation program because of experiencing the intervention as confronting, overwhelming, and deflating [24]. Participants’ experiences may potentially reflect a *sealing over* recovery style, whereby individuals attempt to alleviate distress through avoidance-based coping strategies [59]. Individuals with a sealing over recovery style typically prefer not to think about their experiences of psychosis and avoid talking about their experiences [60]. Congruently, participants who found the website content confronting reported preferring not to think about their mental illness and avoiding engaging with material on this topic. A potential sealing over recovery style was also identified as a reason for wanting to discontinue using the mobile monitoring app in a recent study by Eisner et al [10].

Although these experiences appear consistent with a sealing over approach, the recovery style of participants did not predict engagement with the website in the primary study [16]. This discrepancy may be the result of the modest reliability of the scale used within the primary study, the low number of participants endorsing a strong sealing over recovery style, or misinterpretation of participants’ experiences. As such, further mixed methods research specifically investigating the potential association between recovery style and engagement with web-based interventions among individuals with psychosis is warranted. Regardless of whether these experiences represent a sealing over recovery style, some participants found website content distressing, which resulted in avoidance. Potentially more individualized or in-person support may be required to work through this type of recovery-focused material for some individuals with psychosis.

### Strengths and Limitations

Previous qualitative research has investigated the acceptability and feasibility of using digital technologies for mental health–related purposes [6,53]. However, there has been limited exploration of user perspectives on factors that influence engagement with web-based resources. This study identified several factors that can inform the design and implementation of future web-based interventions. This study may serve as a useful starting point for future research to further investigate processes associated with engagement and outcome in web-based interventions for psychosis. A key strength of this study was the examination of actual, rather than hypothetical, user experiences of the intervention [13]. Methodological integrity was preserved through reflective practice, consultation with other academics, and member checking of results via consultation with individuals with lived experience of psychosis.

We aimed to conduct interviews with participants as soon as possible following the 12-week intervention period. However, as some participants only used the website once or a few times, several weeks had passed between final use and the interview. The interviews were conducted via telephone, which may have potentially limited the richness of collected data [61,62]. Additionally, although we aimed to examine engagement with the website in a naturalistic manner, there may have been unintended effects of the research context that may impact the results [26]. Participants had all agreed to participate in a study involving the use of a web-based intervention and may represent a subgroup of individuals with psychosis who have more interest in digital mental health. In this study, the majority of participants were confident and frequent users of the internet. However, our sample was otherwise sufficiently diverse with regard to gender, age, and level of intervention use.

### Conclusions

Individuals with psychosis experienced several challenges to engagement with a self-guided web-based intervention. In addition to mental health–related problems, psychosocial stressors and navigation issues presented as difficulties engaging with the intervention. Web-based interventions may need to be flexible to accommodate changeable circumstances. The provision of email support may serve as a successful reminder to use an intervention, encourage exploration of intervention aspects, and maintain motivation. Tailoring the intervention to the user’s interests and preferences appears to be important to promote engagement. Further research is required to investigate the relationship between engagement and clinical benefits among individuals with psychosis.

## Appendix

Multimedia Appendix 1 Qualitative interview guide.

## Abbreviations

SMART: Self-Management and Recovery Technology

## References

[1] Gay K, Torous J, Joseph A, Pandya A, Duckworth K (2016). Digital technology use among individuals with schizophrenia: results of an online survey. JMIR Ment Health.

[2] Robotham D, Satkunanathan S, Doughty L, Wykes T (2016). Do we still have a digital divide in mental health? A five-year survey follow-up. J Med Internet Res.

[3] Fernández-Sotos P, Fernández-Caballero A, González P, Aparicio AI, Martínez-Gras I, Torio I, Dompablo M, García-Fernández L, Santos JL, Rodriguez-Jimenez R (2019). Digital technology for internet access by patients with early-stage schizophrenia in Spain: multicenter research study. J Med Internet Res.

[4] Villagonzalo K, Arnold C, Farhall J, Rossell SL, Foley F, Thomas N (2019). Predictors of overall and mental health-related internet use in adults with psychosis. Psychiatry Res.

[5] Thomas N, Foley F, Lindblom K, Lee S (2017). Are people with severe mental illness ready for online interventions? Access and use of the internet in Australian mental health service users. Australas Psychiatry.

[6] Bucci S, Morris R, Berry K, Berry N, Haddock G, Barrowclough C, Lewis S, Edge D (2018). Early psychosis service user views on digital technology: qualitative analysis. JMIR Ment Health.

[7] Firth J, Cotter J, Torous J, Bucci S, Firth JA, Yung AR (2016). Mobile phone ownership and endorsement of 'mhealth' among people with psychosis: a meta-analysis of cross-sectional studies. Schizophr Bull.

[8] Ben-Zeev D, Kaiser SM, Brenner CJ, Begale M, Duffecy J, Mohr DC (2013). Development and usability testing of FOCUS: a smartphone system for self-management of schizophrenia. Psychiatr Rehabil J.

[9] Bucci S, Barrowclough C, Ainsworth J, Machin M, Morris R, Berry K, Emsley R, Lewis S, Edge D, Buchan I, Haddock G (2018). Actissist: proof-of-concept trial of a theory-driven digital intervention for psychosis. Schizophr Bull.

[10] Eisner E, Drake RJ, Berry N, Barrowclough C, Emsley R, Machin M, Bucci S (2019). Development and long-term acceptability of ExPRESS, a mobile phone app to monitor basic symptoms and early signs of psychosis relapse. JMIR Mhealth Uhealth.

[11] Moritz S, Schröder J, Klein JP, Lincoln TM, Andreou C, Fischer A, Arlt S (2016). Effects of online intervention for depression on mood and positive symptoms in schizophrenia. Schizophr Res.

[12] Ben-Zeev D, Brian RM, Jonathan G, Razzano L, Pashka N, Carpenter-Song E, Drake RE, Scherer EA (2018). Mobile health (mhealth) versus clinic-based group intervention for people with serious mental illness: a randomized controlled trial. Psychiatr Serv.

[13] Berry N, Lobban F, Emsley R, Bucci S (2016). Acceptability of interventions delivered online and through mobile phones for people who experience severe mental health problems: a systematic review. J Med Internet Res.

[14] Schlosser DA, Campellone TR, Truong B, Etter K, Vergani S, Komaiko K, Vinogradov S (2018). Efficacy of PRIME, a mobile app intervention designed to improve motivation in young people with schizophrenia. Schizophr Bull.

[15] Naslund JA, Aschbrenner KA, Barre LK, Bartels SJ (2015). Feasibility of popular m-health technologies for activity tracking among individuals with serious mental illness. Telemed J E Health.

[16] Arnold C, Villagonzalo K, Meyer D, Farhall J, Foley F, Kyrios M, Thomas N (2019). Predicting engagement with an online psychosocial intervention for psychosis: exploring individual- and intervention-level predictors. Internet Interv.

[17] Torous J, Staples P, Slaters L, Adams J, Sandoval L, Onnela JP, Keshavan M (2017). Characterizing smartphone engagement for schizophrenia: results of a naturalist mobile health study. Clin Schizophr Relat Psychoses.

[18] Hatch A, Hoffman JE, Ross R, Docherty JP (2018). Expert consensus survey on digital health tools for patients with serious mental illness: optimizing for user characteristics and user support. JMIR Ment Health.

[19] Baup H, Verdoux H (2017). Frequency and pattern of internet use in patients with schizophrenia or bipolar disorders seeking medical information. Psychiatry Res.

[20] Michie S, Yardley L, West R, Patrick K, Greaves F (2017). Developing and evaluating digital interventions to promote behavior change in health and health care: recommendations resulting from an international workshop. J Med Internet Res.

[21] Donkin L, Glozier N (2012). Motivators and motivations to persist with online psychological interventions: a qualitative study of treatment completers. J Med Internet Res.

[22] Poole R, Simpson SA, Smith DJ (2012). Internet-based psychoeducation for bipolar disorder: a qualitative analysis of feasibility, acceptability and impact. BMC Psychiatry.

[23] Johansson O, Michel T, Andersson G, Paxling B (2015). Experiences of non-adherence to internet-delivered cognitive behavior therapy: a qualitative study. Internet Interv.

[24] Nicholas J, Proudfoot J, Parker G, Gillis I, Burckhardt R, Manicavasagar V, Smith M (2010). The ins and outs of an online bipolar education program: a study of program attrition. J Med Internet Res.

[25] Simblett S, Matcham F, Siddi S, Bulgari V, di San Pietro CB, López JH, Ferrão J, Polhemus A, Haro JM, de Girolamo G, Gamble P, Eriksson H, Hotopf M, Wykes T, RADAR-CNS Consortium (2019). Barriers to and facilitators of engagement with mhealth technology for remote measurement and management of depression: qualitative analysis. JMIR Mhealth Uhealth.

[26] Gerhards SA, Abma TA, Arntz A, de Graaf LE, Evers SM, Huibers MJ, Widdershoven GA (2011). Improving adherence and effectiveness of computerised cognitive behavioural therapy without support for depression: a qualitative study on patient experiences. J Affect Disord.

[27] Wilhelmsen M, Lillevoll K, Risør MB, Høifødt R, Johansen M, Waterloo K, Eisemann M, Kolstrup N (2013). Motivation to persist with internet-based cognitive behavioural treatment using blended care: a qualitative study. BMC Psychiatry.

[28] Palmier-Claus JE, Rogers A, Ainsworth J, Machin M, Barrowclough C, Laverty L, Barkus E, Kapur S, Wykes T, Lewis SW (2013). Integrating mobile-phone based assessment for psychosis into people's everyday lives and clinical care: a qualitative study. BMC Psychiatry.

[29] Jonathan G, Carpenter-Song EA, Brian RM, Ben-Zeev D (2019). Life with FOCUS: a qualitative evaluation of the impact of a smartphone intervention on people with serious mental illness. Psychiatr Rehabil J.

[30] Forchuk C, Reiss JP, O'Regan T, Ethridge P, Donelle L, Rudnick A (2015). Client perceptions of the mental health engagement network: a qualitative analysis of an electronic personal health record. BMC Psychiatry.

[31] Thomas N, Farhall J, Foley F, Leitan ND, Villagonzalo K, Ladd E, Nunan C, Farnan S, Frankish R, Smark T, Rossell SL, Sterling L, Murray G, Castle DJ, Kyrios M (2016). Promoting personal recovery in people with persisting psychotic disorders: development and pilot study of a novel digital intervention. Front Psychiatry.

[32] Thomas N, Farhall J, Foley F, Rossell SL, Castle D, Ladd E, Meyer D, Mihalopoulos C, Leitan N, Nunan C, Frankish R, Smark T, Farnan S, McLeod B, Sterling L, Murray G, Fossey E, Brophy L, Kyrios M (2016). Randomised controlled trial of a digitally assisted low intensity intervention to promote personal recovery in persisting psychosis: SMART-therapy study protocol. BMC Psychiatry.

[33] Anthony WA (1993). Recovery from mental illness: the guiding vision of the mental health service system in the 1990s. Psychiatr Rehabil J.

[34] Rotondi AJ, Sinkule J, Haas GL, Spring MB, Litschge CM, Newhill CE, Ganguli R, Anderson CM (2007). Designing websites for persons with cognitive deficits: design and usability of a psychoeducational intervention for persons with severe mental illness. Psychol Serv.

[35] Drake RE, Whitley R (2014). Recovery and severe mental illness: description and analysis. Can J Psychiatry.

[36] Braun V, Clarke V (2006). Using thematic analysis in psychology. Qual Res Psychol.

[37] Barriball KL, While A (1994). Collecting data using a semi-structured interview: a discussion paper. J Adv Nurs.

[38] Reid K, Flowers P, Larkin M (2005). Exploring lived experience. Psychologist.

[39] Madill A, Given LM (2008). Realism. The Sage Encyclopedia of Qualitative Research Methods.

[40] First M, Spitzer R, Gibbon M, Williams J (2002). User's Guide for the Structured Clinical Interview for Dsm-IV Axis I Disorders: Scid-1 Clinician Version.

[41] Levitt HM, Bamberg M, Creswell JW, Frost DM, Josselson R, Suárez-Orozco C (2018). Journal article reporting standards for qualitative primary, qualitative meta-analytic, and mixed methods research in psychology: the APA publications and communications board task force report. Am Psychol.

[42] Bendelin N, Hesser H, Dahl J, Carlbring P, Nelson KZ, Andersson G (2011). Experiences of guided internet-based cognitive-behavioural treatment for depression: a qualitative study. BMC Psychiatry.

[43] Mohr DC, Cuijpers P, Lehman K (2011). Supportive accountability: a model for providing human support to enhance adherence to ehealth interventions. J Med Internet Res.

[44] Nicholas J, Fogarty AS, Boydell K, Christensen H (2017). The reviews are in: a qualitative content analysis of consumer perspectives on apps for bipolar disorder. J Med Internet Res.

[45] Titov N, Dear BF, Johnston L, Lorian C, Zou J, Wootton B, Spence J, McEvoy PM, Rapee RM (2013). Improving adherence and clinical outcomes in self-guided internet treatment for anxiety and depression: randomised controlled trial. PLoS One.

[46] Ryan RM, Deci EL (2000). Intrinsic and extrinsic motivations: classic definitions and new directions. Contemp Educ Psychol.

[47] Pung A, Fletcher SL, Gunn JM (2018). Mobile app use by primary care patients to manage their depressive symptoms: qualitative study. J Med Internet Res.

[48] Morrison LG, Yardley L, Powell J, Michie S (2012). What design features are used in effective e-health interventions? A review using techniques from critical interpretive synthesis. Telemed J E Health.

[49] Yardley L, Spring BJ, Riper H, Morrison LG, Crane DH, Curtis K, Merchant GC, Naughton F, Blandford A (2016). Understanding and promoting effective engagement with digital behavior change interventions. Am J Prev Med.

[50] Yardley L, Morrison L, Bradbury K, Muller I (2015). The person-based approach to intervention development: application to digital health-related behavior change interventions. J Med Internet Res.

[51] Seed T, Fox JR, Berry K (2016). The experience of involuntary detention in acute psychiatric care. A review and synthesis of qualitative studies. Int J Nurs Stud.

[52] Mohr DC, Schueller SM, Montague E, Burns MN, Rashidi P (2014). The behavioral intervention technology model: an integrated conceptual and technological framework for ehealth and mhealth interventions. J Med Internet Res.

[53] Aref-Adib G, O'Hanlon P, Fullarton K, Morant N, Sommerlad A, Johnson S, Osborn D (2016). A qualitative study of online mental health information seeking behaviour by those with psychosis. BMC Psychiatry.

[54] Walsh A, Richards D (2016). Experiences and engagement with the design features and strategies of an internet-delivered treatment programme for generalised anxiety disorder: a service-based evaluation. Brit J Guid Couns.

[55] Agar-Jacomb K, Read J (2009). Mental health crisis services: what do service users need when in crisis?. J Ment Health.

[56] Rotondi AJ, Eack SM, Hanusa BH, Spring MB, Haas GL (2015). Critical design elements of e-health applications for users with severe mental illness: singular focus, simple architecture, prominent contents, explicit navigation, and inclusive hyperlinks. Schizophr Bull.

[57] Mohr DC, Weingardt KR, Reddy M, Schueller SM (2017). Three problems with current digital mental health research . . . and three things we can do about them. Psychiatr Serv.

[58] Depp C, Perivoliotis D, Holden J, Dorr J, Granholm E (2019). Single-session mobile-augmented intervention in serious mental illness: a three-arm randomized controlled trial. Schizophr Bull.

[59] McGlashan TH, Levy ST, Carpenter WT (1975). Integration and sealing over. Clinically distinct recovery styles from schizophrenia. Arch Gen Psychiatry.

[60] McGlashan TH, Docherty JP, Siris S (1976). Integrative and sealing-over recoveries from schizophrenia: distinguishing case studies. Psychiatry.

[61] Heath J, Williamson H, Williams L, Harcourt D (2018). 'It's just more personal': using multiple methods of qualitative data collection to facilitate participation in research focusing on sensitive subjects. Appl Nurs Res.

[62] Johnson DR, Scheitle CP, Ecklund EH (2019). Beyond the in-person interview? How interview quality varies across in-person, telephone, and Skype interviews. Soc Sci Comput Rev.

