# Supplementary material for: Engaging With a Web-Based Psychosocial Intervention for Psychosis: Qualitative Study of User Experiences
Source: JMIR Ment Health. 2020 Jun 19;7(6):e16730. doi: 10.2196/16730 (PMC7334758; doi:10.2196/16730)
Supplement: Multimedia Appendix 1 [file mental_v7i6e16730_app1.docx]

**Qualitative Interview Guide**

**Focus 1 -** General experience of using self-guided digital resource

- *How did you find using the website (by yourself?)*
- *Can you tell me about a time that:*
  - *You used the website by yourself*
    - Probes: Time, place, device used, alone/with someone?
  - *Something prevented you from using the website*

**Focus 2 -** Factors influencing use of the resource

- *What made you start using SMART? (What got you going with SMART at the beginning?)*
  - Recruitment process?
- *What, if anything, helped you continue using SMART after you started?*
  - Probe: Process
- *What, if anything, prevented you from using (or continuing to use) the website?*
  - Probe: Process
- *What, if anything, made the website easy to continue using?*
- *What, if anything, made it difficult to continue using the website?*
- *What, if anything, made you stop using the website?*

Explore pattern of use + whether this changed over time.

**Focus 3 -** Email support and therapeutic relationship

Email Group:

- *How did you find receiving the weekly emails?*
  - Probes: frequency, medium (email v phone), improvements?
- *What difference, if any, do you think receiving the emails made to your experience of using the website?*
- *Imagine if… [other condition], how do you think that might have influenced your experience, if at all?*
- *How was the relationship between you and your online therapist?*
  - *Did your relationship influence your (experience of) use of the website in any way?*

Website only group:

- *How did you find working through the material by yourself?*
- *What difference, if any, do you think getting extra support would have made to your experience of using the website?*
- *Imagine if… [other condition], how do you think that might have influenced your experience, if at all?*
- *What types of support might you recommend?*

**Focus 4 -** Website features (and influence on use)

- *Were there any parts of the website that you came back to?*
- *Did those features have any influence on your use of the site? Or experience of using the website?*
- *Were there any parts you preferred not to use?*

**Focus 5** – Engagement with content outside website use

- *Did you have specific goals related to using the website?*
- *Have you noticed any changes in your life since you started using the website?*
- *What do you think may have influenced those changes?*
  - Probe: processes relating to website content?
